# Patient perceptions of an electronic-health-record-based rheumatoid arthritis outcomes dashboard: a mixed-methods study

**DOI:** 10.1186/s12911-024-02696-9

**Published:** 2024-10-12

**Authors:** Catherine Nasrallah, Cherish Wilson, Alicia Hamblin, Christine Hariz, Cammie Young, Jing Li, Jinoos Yazdany, Gabriela Schmajuk

**Affiliations:** 1 Division of Rheumatology, Department of Medicine, University of California, 4150 Clement Street, Mailstop 111R, San Francisco, CA 94121 USA; 2 San Francisco Veterans Affairs Medical Center, San Francisco, CA USA; 3 Center for Vulnerable Populations and Zuckerberg San Francisco General Hospital, San Francisco, CA USA

**Keywords:** Rheumatoid Arthritis, Patient Reported Outcomes, Dashboard, Qualitative interviews, Patients, Perceptions, Ecological Model of Health, Mixed-Methods, Disease Activity, Physical Function

## Abstract

**Background:**

Outcome measures are crucial to support a treat-to-target approach to rheumatoid arthritis (RA) care, yet their integration into clinical practice remains inconsistent. We developed an Electronic Heath Record-integrated, patient-facing side-car application to display RA outcomes (disease activity, functional status, pain scores), medications, and lab results during clinical visits (“RA PRO Dashboard”). The study aimed to evaluate patient perceptions and attitudes towards the implementation of a novel patient-facing dashboard during clinical visits using a mixed-methods approach.

**Methods:**

RA patients whose clinicians used the dashboard at least once during their clinical visit were invited to complete a survey regarding its usefulness in care. We also conducted semi-structured interviews with a subset of patients to assess their perceptions of the dashboard. The interviews were transcribed verbatim and analyzed thematically using deductive and inductive techniques. Emerging themes and subthemes were organized into four domains of the Ecological Model of Health.

**Results:**

Out of 173 survey respondents, 79% were interested in seeing the dashboard again at a future visit, 71% felt it improved their understanding of their disease, and 65% believed it helped with decision-making about their RA care. Many patients reported that the dashboard helped them discuss their RA symptoms (76%) and medications (72%) with their clinician. Interviews with 29 RA patients revealed 10 key themes: the dashboard was perceived as a valuable visual tool that improved patients’ understanding of RA outcome measures, enhanced their involvement in care, and increased their trust in clinicians and the clinic. Common reported limitations included concerns about reliability of RA outcome questionnaires for some RA patients and inconsistent collection and explanation of these measures by clinicians.

**Conclusions:**

In both the quantitative and qualitative components of the study, patients reported that the dashboard improved their understanding of their RA, enhanced patient-clinician communication, supported shared decision-making, and increased patient engagement in care. These findings support the use of dashboards or similar data visualization tools in RA care and can be used in future interventions to address challenges in data collection and patient education.

**Supplementary Information:**

The online version contains supplementary material available at 10.1186/s12911-024-02696-9.

## Background

Shared decision-making, health literacy, and effective communication around outcome measures are important components of a treat-to-target approach in rheumatoid arthritis (RA) [1–3], a chronic autoimmune disease characterized by significant pain, joint swelling and stiffness, inflammation, and fatigue [4]. Standardized RA outcome measures provide reliable indicators of patients’ experiences of their disease activity (DA), functional status, symptoms, and pain levels over time [3, 5]. Three outcome measures have been identified as part of an RA “core set”: 1) Patient global assessment of disease utilized as a component of a composite DA measure, 2) Pain, and 3) Physical function (PF) [6]. Thus, routine collection and assessment of these outcomes is recommended by the American College of Rheumatology (ACR) to monitor changes over time, improve patient outcomes, guide clinical decision making, and promote patient-centered care [7–10].

Despite recommendations for regular collection of RA outcome measures and their proven benefits for patient-clinician communication and patient health outcomes, use of these measures is inconsistent in clinical care [11–14]. A few studies have reported on perceived barriers to the integration of RA outcome measures into clinical practice by clinicians, including concerns about the complexity of outcome measure questionnaires, and the belief that incorporation of these measures might hinder interactions with patients [15] or detract from a patient-focused encounter [16].

Several studies have shown that use of electronic dashboards that display outcome measures during clinical visits positively impacted shared decision-making, improved collection and integration of patient reported outcomes (PROs) into the clinical workflow, enhanced symptom control, and increased health-related quality of life [17–20]. In rheumatology, several dashboards have been developed to help improve quality of care [21, 22] and the DANBIO register in Denmark features a dashboard for clinical practice that has been utilized to collect PROs from patients and track RA outcomes over time [23]. However, these dashboards were primarily designed for clinician use, as opposed to being patient-facing. A recent study described adaptations made to the EPIC native rheumatology module to implement a patient-facing visualization that tracked PROs for patients with RA and juvenile idiopathic arthritis [24]: this dashboard helped users make sense of health information, communicate more effectively, and make decisions.

At our institution, we recently used a human-centered design process to develop and deploy an Electronic Heath Record (EHR)-integrated, patient-facing side-car application to display RA outcomes (disease activity, functional status, pain scores), medications, and lab results during clinical visits (“RA PRO Dashboard”). The dashboard was designed to be patient-facing and customizable in its display of data [25]. In this study, we aimed to evaluate patient perceptions and attitudes around RA outcomes and the implementation of this novel patient-facing dashboard using a mixed-methods approach guided by the Ecological Model of Health (EMH) framework. Roll-out of the dashboard provided a unique opportunity to query patients about the dashboard interface and about RA outcome measures more generally. To our knowledge, this is among the first studies to focus on patient perspectives on the benefits and limitations of using a digital health tool displaying RA outcome measures in clinical practice.

## Methods

### RA PRO dashboard development

**Fig. 1:**
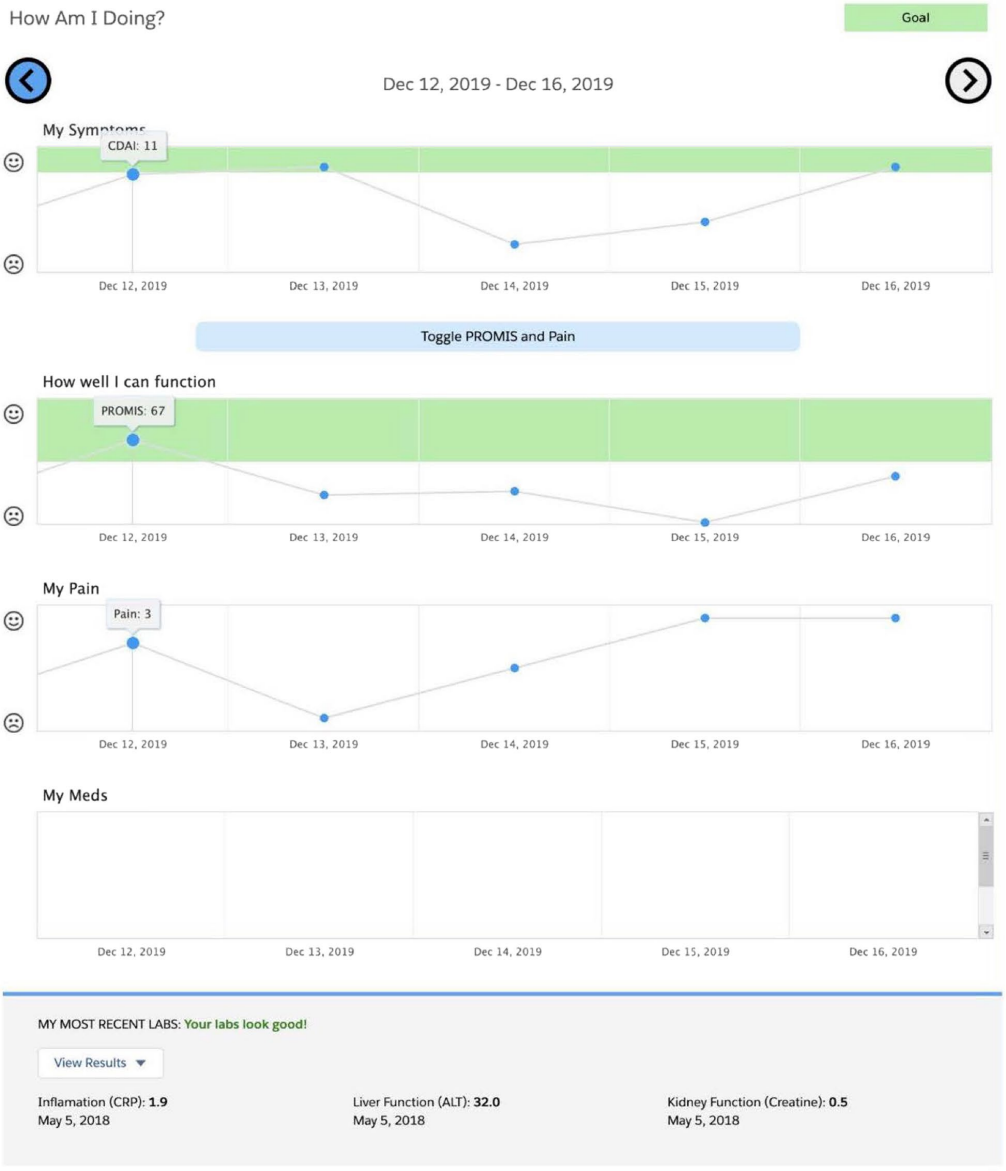
Screenshot of the RA PRO dashboard. Nasrallah C, Wilson C, Hamblin A, Young C, Jacobsohn L, Nakamura MC, Gross A, Matloubian M, Ashouri J, Yazdany J, Schmajuk G. Using the technology acceptance model to assess clinician perceptions and experiences with a rheumatoid arthritis outcomes dashboard: qualitative study. BMC Med Inform Decis Mak. 2024 May 27;24(1):140. 10.1186/s12911-024-02530-2. PMID: 38802865; PMCID: PMC11129391

We used principles of human-centered design [26, 27] to develop an EHR-integrated, patient-facing sidecar RA PRO dashboard application. As described previously, the dashboard pulls RA outcome data from the EHR that are collected routinely during clinical visits (CDAI, PROMIS-PF and pain) and displays graphs showing their trajectories over time, including established clinical targets, as well as medications and lab results (Fig. 1) [25]. The data shown on the dashboard are derived from existing structured fields in the EHR. Data from 2014 onwards are included (as are values from the same day’s visit). The dashboard is designed to be shared by clinicians with patients, either on the computer screen during in-person clinical visits or using a share-screen function during telehealth visits.

### Clinical setting

The dashboard was implemented at an academic rheumatology clinic in northern California which serves approximately 500 patients with RA. The clinic is staffed by a rheumatology nurse practitioner and approximately 12 attending physicians who see patients between 1 and 4 half-days per week. Attendings also supervise 6 rheumatology trainees, who rotate through the clinic for 1–3 years.

Patient-reported components of RA outcome measures are typically collected by medical assistants (MAs) when the patient checks in for their in-person visit, or when they initially log on to a telehealth visit. Patients are asked to complete 1) a pain question to assess arthritis pain over the past seven days using a visual-analog scale of 0–100, 2) the PROMIS-PF questionnaire to assess PF, and 3) the patient global assessment of RA activity, which is used by clinicians to calculate the CDAI. After completing the forms, MAs input the raw scores into structured fields in the EHR, whereby the PROMIS-PF scores are converted into *t*-scores [28]. Once the clinician enters the evaluator global assessment and tender and swollen joint counts, the EHR uses the patient global assessment score to generate the DA score as part of the CDAI [29]. Adherence to these workflows is routine: PROMIS-PF and pain scores are documented in the EHR at nearly 80% of visits (both in-person and telehealth); CDAIs are captured at approximately 60% of in-person visits.

The RA PRO dashboard went live in August 2021 as part of a stepped-wedge pragmatic cluster-randomized trial. Clinicians were trained by study coordinators (CW, AH, CH, CY) on how to navigate the dashboard. Clinicians could engage with the dashboard or share it with their patients at their own discretion. During our study period (August 2021 through April 2023), 19.8% of RA visits had confirmed interactions with the dashboard, representing 318 unique patients who saw the dashboard at least one time during this period.

### Study design

This study used a mixed-methods approach to evaluate the patients’ experiences, acceptability, and perceived usefulness of the RA PRO dashboard shared by their clinician during their visit. By combining quantitative and qualitative data, we aimed to gain a thorough understanding of patient perspectives and identify any suggestions for potential improvements to the dashboard. This approach allowed us to complete a robust analysis that compared numerical survey results with detailed patient feedback.

### Data collection

#### Quantitative data collection

Eligibility criteria for participation in the patient surveys required that patients be ≥ 18 years of age, with ≥ 2 International Classification of Diseases (ICD) codes for RA at least 30 days apart, receiving care at the academic rheumatology clinic, proficient in English (primary spoken language documented as English in the EHR), and had seen the dashboard during their most recent rheumatology clinic visit. The inclusion criteria ensured that patients had direct experience with the dashboard prior to completing the survey. All patients who saw the RA PRO dashboard during the study period were invited to participate in the quantitative survey (*n* = 318).

Patients who were interested in participating in the survey were explained the aim of the study and verbally consented to participate. The survey included statements regarding patients’ experiences with the RA PRO dashboard, evaluating its acceptability and usefulness during the visit, and their willingness to see it in future visits, using a 4-point Likert-type scale (“Yes", “Somewhat”, “No”, “Unsure”; see specific survey items in Table 2). We included a balancing question: “Do you think using the dashboard changed the focus of your visit?” to understand whether using the RA PRO dashboard during the clinic visit may have adversely affected the patient-clinician interaction during the visit (e.g., shifted the focus from the patient to the computer). Therefore, this question was an “inverse” measure where a “No” response reflected positive impressions of the dashboard. Patients could complete the survey more than once if they had multiple visits in the clinic during the study period.

#### Qualitative data collection

Eligibility criteria for patient interviews required patients had completed at least one quantitative survey above. Purposive sampling was used to ensure that a range of usability perspectives were captured (i.e., patient quantitative survey responses were varied, and that patients were cared for by different clinicians in the clinic) [30].

Semi-structured interviews were conducted with a subset of patients who completed at least one dashboard survey (discussed above) aiming to gather in-depth insights into patients' experiences with the RA PRO dashboard and perceptions towards its usefulness and usability. To ensure that a range of usability perspectives were included, we invited 141 patients treated by various clinicians to participate in these interviews. First, study team members (CW, AH, CH, CY) reached out to eligible patients in-person at the clinic or by phone after their telehealth visit with their clinician and explained the purpose of the study, its voluntary nature, and asked them to contact the research team if interested in scheduling an interview. An experienced researcher trained in qualitative research methods (CN) conducted virtual interviews with patients via Zoom between November 2022 and April 2023 and team members (CW, CY) took notes. The interviews were guided by a semi-structured interview guide developed by CN (Additional File 1) that assessed patients’ perceptions towards RA outcome measures and explored their experience with the RA PRO dashboard shared by their clinician during their visit. The interview guide was tested with two rheumatologists (JY, GS), and piloted with the first few patients included in the study to ensure that the questions were understandable and relevant to end users. No major changes were required as a result of this testing. Interviews lasted between 20 to 45 min and were audio-recorded and transcribed verbatim. Later, transcripts were checked against recordings for quality assurance. Anonymity of patients was protected through de-identifying the transcripts. Study activities were approved by the Institutional Review Board at the University of California, San Francisco.

### Theoretical framework

Our analysis was guided by the EMH, which provides a framework to assess multiple factors at the patient, interpersonal, clinician, and clinic levels that might influence patient perceptions of RA outcome measures and their experience with the RA PRO dashboard [31]. The EMH framework addresses the complexities, interactions, and interdependencies between individual, interpersonal, and organizational determinants of health. It has been widely used in public health settings to explore patient preferences, perceptions, and attitudes towards different health matters, to identify modifiable factors at the appropriate level, and ultimately to design targeted interventions to improve clinical inertia.

### Analysis

#### Quantitative analysis

Survey data were analyzed descriptively using STATA 18 [32] including frequencies and mean scores for scale items. Results were organized as per the levels of the EMH, excluding the clinical level domain since survey responses did not capture information for that domain. For participants that completed more than one survey over the study period (*n* = 57), only their initial survey response was included in the primary analysis. A separate table was generated showing responses of the most recent survey for the 57 patients with more than one survey collected.

#### Qualitative analysis

**Fig. 2:**
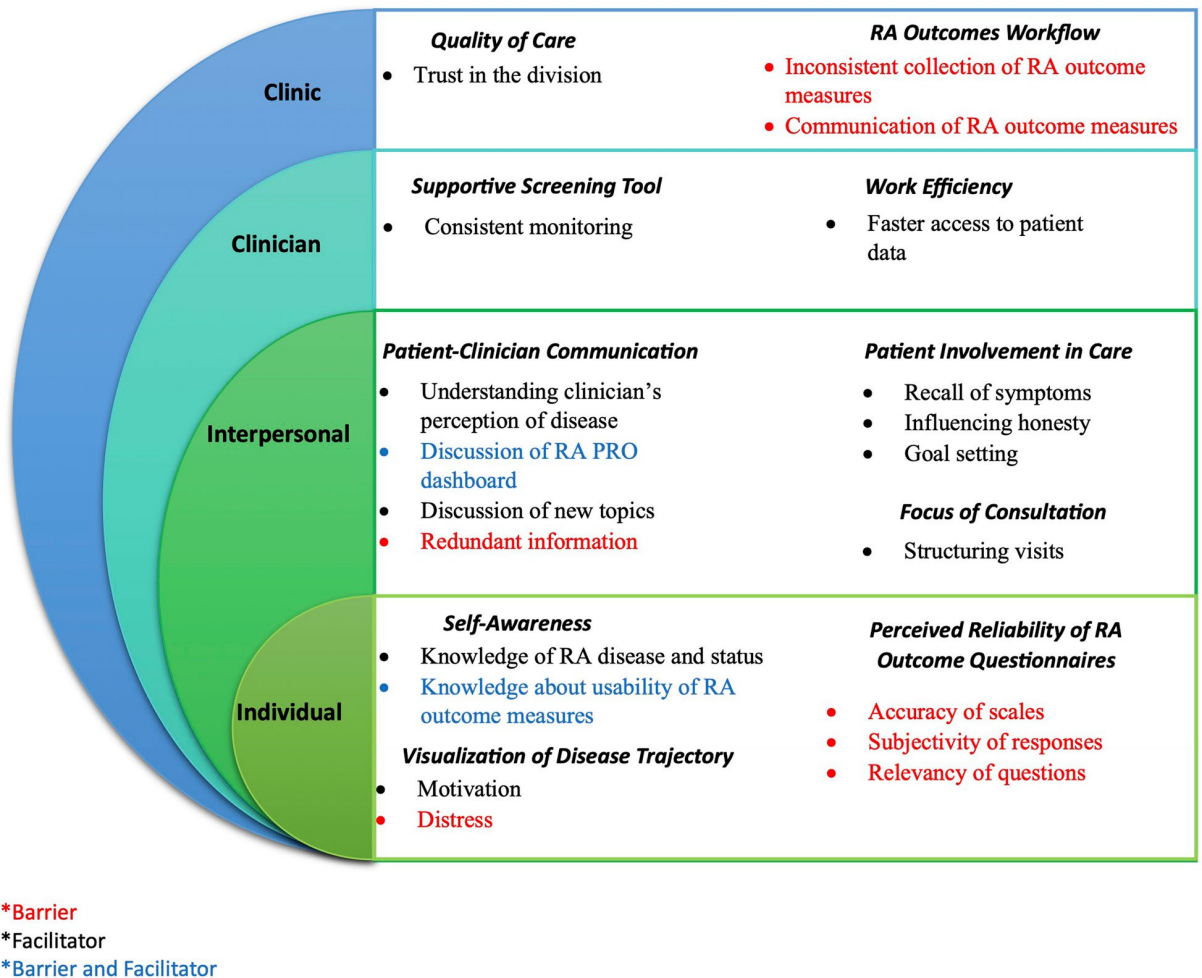
Generated themes and sub-themes as the ecological model

Responses to open-ended interview questions were analyzed thematically using deductive and inductive techniques to identify key themes and subthemes [33]. Open-ended questions allowed respondents to elaborate on specific aspects of their experiences with the RA PRO dashboard, capturing nuanced feedback that complimented the quantitative data. Using Dedoose version 7.0.23 [34], an experienced qualitative researcher (CN) reviewed the transcripts to apply a set of deductive codes based on the EMH and created a preliminary set of inductive codes to capture emergent ideas within and across the interviews. Applied codes were later discussed, edited, and organized into a structured codebook with definitions for each code. Three members of the study team (CW, AH, CH) independently applied the codes to each transcript (2 coders per transcript) and discrepancies were resolved via consensus meetings with a fourth coder (CN). Data were then reviewed by deductive and inductive codes to identify emerging themes and subthemes, which were organized into the domains of the EMH using a systematic and iterative process [35] (Fig. 2). Then, all coded excerpts were summarized and sorted by their relevant themes and subthemes with quotes illustrating how each theme or subtheme served as a benefit or disadvantage of RA outcome measures in general and the RA PRO dashboard in specific as perceived by the patients. The study complied with the Consolidated Criteria for reporting Qualitative Research (COREQ) checklist (Additional File 2).

## Results

### Survey findings

A total of 173 patients completed the survey. Participant demographics are summarized in Table 1. Responses to survey items are shown in Table 2. Visits preceding completion of the questionnaires were mostly in-person (89%). Respondents had visits with 15 different clinicians, although the majority saw one of six clinicians (Additional File 3).

**Table 1:** Socio-demographic characteristics of study participants

	**Patient Survey Respondents (*N* = 173)**	**Patient Interview Participants (*n* = 29)**
**Age, mean (SD), n (%)**	57.9 (14.3)	57.8 (15.0)
< 30	4 (2.3)	-
30–44	29 (16.8)	9 (31.0)
45–64	63 (36.4)	5 (17.2)
65–74	52 (30.0)	14 (48.3)
≥ 75	25 (14.5)	1 (3.4)
**Gender, n (%)**		
Female	150 (86.7)	25 (86.2)
Male	23 (13.3)	4 (13.8)
**Race and Ethnicity,** **n (%)**		
African American or Black	17 (9.8)	1 (3.4)
Asian	32 (18.5)	8 (27.6)
Hispanic	23 (13.3)	2 (6.9)
Mixed/Other	8 (4.6)	-
White	90 (52.0)	17 (58.6)
Unknown	3 (1.7)	1 (3.4)
**Insurance,** **n (%)**		
Medicaid	18 (10.4)	5 (17.2)
Medicare	87 (50.3)	16 (55.2)
Private/Commercial	68 (39.3)	8 (27.6)
**Baseline Comorbidities**^a^, **n (%)**		
Anxiety	24 (13.9)	5 (17.2)
Depression	21 (12.1)	5 (17.2)
Fibromyalgia	9 (5.2)	0 (0)
**Baseline RA Outcomes**^b^		
CDAI, n	163	26
median (IQR)	9.2 (4.5—17.4)	6.4 (3.5–15.0)
PROMIS-PF 10a (T score), n	154	25
median (IQR)	41.8 (35.7—49.1)	47 (37.7—51.3)
Pain, n	145	22
median (IQR)	34 (13.0—60.0)	21 (5.0—39.0)
**Highest Level of Education,** **n (%)**	Not available	
Graduate Degree (Master's or Doctoral Degree)	-	13 (44.8)
College Graduate (bachelor’s degree)	-	10 (34.5)
Some College or Technical School	-	4 (13.8)
High School Graduate or G.E.D	-	1 (3.4)
Less than High School	-	1 (3.4)
**Employment Status,** **n (%)**	Not available	
Retired or disabled	-	10 (34.5)
Full-time employee	-	5 (17.2)
Homemaker	-	4 (13.8)
Unemployed, not looking for a job	-	4 (13.8)
Unemployed, looking for a job	-	2 (6.9)
Part-time employee	-	2 (6.9)
Student, unemployed	-	1 (3.4)
Student, employed	-	1 (3.4)
**How confident are you filling out medical forms by yourself?** **n (%)**	Not available	
Extremely	-	22 (75.9)
Quite a Bit	-	6 (20.7)
Somewhat	-	1 (3.4)

^a^Baseline Comorbidities defined as 1 ICD code within 12 months prior to their clinic visit date

^b^Baseline RA Outcomes were the most recent RA outcome within 18 months to their clinic visit date

**Table 2:** Summary of survey responses, categorized according to the Ecological Model of Health (*N* = 173)

**INDIVIDUAL, n (%)**	**Yes**	**Somewhat**	**No**	**Missing/** **Unsure**
Would you like to see the dashboard again at your next visit?	136 (78.6)	-	14 (8.1)	23 (13.3)
Did the dashboard help you understand more about your RA?	122 (70.5)	35 (20.2)	9 (5.2)	7 (4.0)
Did the dashboard help you understand more about why you take certain medicines?	103 (59.5)	26 (15.0)	32 (18.5)	12 (6.9)
Did the dashboard help you share information about your RA with other people (such as family members, friends, or other healthcare providers)?	74 (42.8)	26 (15.0)	41 (23.7)	32 (18.5)
**INTERPERSONAL, n (%)**	**Yes**	**Somewhat**	**No**	**Missing/** **Unsure**
Did the dashboard help you talk to your doctor about your RA or your symptoms?	131 (75.7)	24 (13.9)	14 (8.1)	4 (2.3)
Did the dashboard help you talk to your doctor about your medicines?	125 (72.3)	22 (12.7)	18 (10.4)	8 (4.6)
Did the dashboard help you make better decisions about your RA care?	112 (64.7)	33 (19.1)	17 (9.8)	11 (6.4)
Did the dashboard help you talk about things that are important to managing your disease, other than your medicines?	107 (61.8)	28 (16.2)	29 (16.8)	9 (5.2)
Do you think using the dashboard helped your communication with your doctor?	97 (56.1)	40 (23.1)	26 (15.0)	10 (5.8)
Do you think using the dashboard changed the focus of your visit?	38 (22.0)	43 (24.9)	77 (44.5)	15 (8.7)
**CLINICIAN, n (%)**	**Yes**	**Somewhat**	**No**	**Missing/** **Unsure**
Do you think using the dashboard helped your doctor to better understand what's most important to you?	79 (45.7)	33 (19.1)	37 (21.4)	24 (13.9)
Do you think using the dashboard gave your doctor information about you that s/he may not have gotten without the dashboard?	65 (37.6)	42 (24.3)	38 (22.0)	28 (16.2)

#### Patient level

Survey responses showed that more than two-thirds of patients (78.6%) wanted to see the dashboard again at a future visit. Regarding individual benefits of the dashboard, 70.5% of patients reported that it helped them understand more about their diseases, more than half (59.5%) understood why they were prescribed certain medications, and slightly less than half (42.8%) agreed that the dashboard helped them share information about their RA with others.

#### Interpersonal level

Three-quarters of patients felt that the dashboard helped them talk to their doctor about their RA or symptoms (75.7%) and medications (72.3%). 61.8% of patients considered that the dashboard helped them talk with their clinician about other things that are important to managing their RA, with slightly more than half of respondents (56.1%) reporting that the dashboard helped their overall patient-clinician communication. A significant majority of patients (64.7%) found the dashboard useful in improving their decision-making regarding RA care. With regards to the balancing question, a minority of patients (22.0%) reported a change in the focus of their clinical visits due to the dashboard.

#### Clinician level

Slightly less than half of patients (45.7%) felt that using the dashboard helped their clinicians better understand what is most important to them, and around a third of respondents (37.6%) considered that using the dashboard gave their clinician additional information that they would otherwise not have had.

Findings from the most recent survey responses among patients with more than one survey completed (*n* = 57) did not differ substantially from the primary analysis (Additional File 4).

### Interview findings

141 patients who completed the quantitative surveys were invited to participate in patient interviews (characteristics in Additional File 5). We ceased patient recruitment and data collection after completing 29 interviews with patients treated by 9 unique physicians and reaching data saturation (later interviews were not bearing new perspectives on RA outcome measures or the RA PRO dashboard). Interview participant demographics are summarized in Table 1. The majority of patients interviewed were well-educated (79.3% had at least a college or graduate degree) and demonstrated a high level of confidence in completing medical forms independently (75.9%). Quantitative survey responses from the 29 patients who completed interviews were similar to those observed in the primary analysis sample of 173 (Additional File 6).

Ten key themes were identified, representing patients’ perceived benefits and limitations of collecting RA outcome measures and experiences with the RA PRO dashboard in clinical practice. Generated themes and subthemes were organized into the four levels of influence according to the EMH: Patient, interpersonal, clinician, and clinic level. Below, we summarize themes and subthemes within each level and provide exemplary quotes.

#### Patient level

Three main themes emerged at the individual patient level: self-awareness, visualizing disease trajectory, and perceived reliability of RA outcome questionnaires. Most patients reported that completing RA outcome questionnaires and discussing changes over time with their clinician by reviewing the RA PRO dashboard increased their understanding of their symptoms and improved their knowledge about their disease prognosis (Table 3.: Q1.1). In terms of knowledge about usability of RA outcome measures, patients’ understanding of the role of these measures in RA care depended on clinicians’ use of the dashboard. For instance, patients whose clinician used the RA PRO dashboard at every clinical visit to discuss updated RA outcome scores expressed high knowledge about the measures collected and understanding of target scores. They stated that the content of the dashboard provided them with “information that they couldn’t understand before” regarding score calculation and interpretation (Table 3.: Q1.2). However, patients whose clinician did not frequently use the dashboard or did not discuss RA outcome measure results showed limited understanding of the role, benefits, and use of these measures in RA care (Table 3.: Q1.3).

**Table 3:** Benefits and limitations of collecting RA outcome measures and using the RA PRO dashboard as per the Ecological Model of Health—Interview quotes

**Levels**	**Themes**	**Subtheme**	***Quotes***
**Patient**	**Self-Awareness**	Knowledge of RA disease and status [Facilitator]	*Q1.1. "Track progression of the disease and symptoms over time, which is helpful since a lot of the times I forget how it's been going for longer than six months ago. I've sort of forgotten how things have been progressing. More of a historical view across my symptoms and overall disease as a whole is interesting to look at." (P630)*
		Knowledge about usability of RA outcome measures [Facilitator and Barrier]	*Q1.2. "I never talked with my rheumatologist before about those scores. I could see them sometimes in my notes, but it didn’t mean anything to me, so it was more interesting. The dashboard made a conversation happen like, 'How is that calculated? What data are going into that to calculate a CDAI score?'” (P186)*
			*Q1.3. "I don't know what PROMIS is. I'm not quite sure what that's based on. I know that I answer some questions at the beginning of the appointment about how I'm functioning, but I'm not sure how that translates to this graph." (P574)*
	**Visualization of Disease Trajectory**	Motivation [Facilitator]	*Q1.4 . "It gave me hope that, although I'm not there yet, I'm much better than a year and a half ago. And that's very important. That really empowered me to feel like, oh, we're taking actions. And, visually, there's proof that we're heading towards recovery." (P437)*
		Distress [Barrier]	*Q1.5. "To be honest, I would say I probably walked out of there more depressed. Because it’s one thing to have sort of a narrative that you’re getting worse. It’s certainly something else to see...where you’re getting worse. And, I mean, yes, I absolutely appreciate the information. I’m not saying I don’t want to see it... But it’s definitely, 'Oh, gee, this is even worse than I thought.'" (P225)*
	**Perceived Reliability of RA Outcome Questionnaires **	Accuracy of scales [Barrier]	*Q1.6. "They give you zero to 100, I remember, just one line... you have no room to measure. You're just eyeballing. Maybe today my mood feels like I'm okay. Maybe I'll do a little bit close to zero. Maybe I'm not feeling good. Maybe I do it in the middle. With that line, I just don't think it's accurate at all, to me." (P715)*
		Subjectivity of responses [Barrier]	*Q1.7. "It's self-reported, right? So, what are your symptoms? And so, I'd say these are the symptoms. And, you know, it's zero to 100 or zero to whatever. And that's pretty subjective from a human point of view." (P370)*
		Relevancy of questions [Barrier]	*Q1.8. "The PROMIS physical function score, that’s based on your answers to questions. And I found some of the questions slightly ambiguous. ‘Could you work physical labor eight hours a day?’ I don’t know what they mean by physical labor. I can do what you are doing right now eight hours a day easy. But could I carry bricks eight hours a day?" (P799)*
**Interpersonal **	**Patient-Clinician Communication**	Understanding clinician’s perception of disease [Facilitator]	*Q2.1. "And then he uses the dashboard in my meeting with him. You know, we go over it, and he says, ‘You know, I think you think you're a two, but I think you're really a five.’ [chuckles] You know, as far as pain goes." (P448)*
		Discussion of RA PRO dashboard [Facilitator and Barrier]	*Q2.2. "They're considering your pain control, your medication, your inflammation, your bloodwork. While I have an understanding of my bloodwork and what that all means, I'm not a scientist. Dr. [Physician’s name] is my rheumatology data guy. I need people on my team to fill in the gaps for things that I don't have education in or understanding of – not a thorough understanding." (P164)*
			*Q2.3. "The doctor didn't really go over it with me.... I wonder why she hasn't shared with me...It's my data." (P802)*
		Discussion of new topics [Facilitator]	*Q2.4. "What I remember her always talking to me about is if I'm in remission or not, because I have small flares, and because of my job and because I overdo it. Or I don't sleep, or I eat too much inflammatory–sugar or whatever." (P471)*
		Redundant information [Barrier]	*Q2.5. "I don't think it added any information to what I always get at his visits... I didn't really get anything from the dashboard that I don't already get from him." (P679) *
	**Patient Involvement in Care**	Recall of symptoms [Facilitator]	* Q2.6. "I like to be able to see, you know, am I doing better in one area than I was? Maybe I have a high activity because I've been stressed, and I have to remember to relax or to exercise." (P574)*
		Influencing honesty [Facilitator]	*Q2.7. "I'm also learning to be more honest about it, to be truthful, because I don't want to be so honest about how much pain I'm in, [chuckles] because then it acknowledges the pain." (P448)*
		Goal setting [Facilitator]	*Q2.8. "For me to make a determination whether to change a medication, I need to know: What does my future look like? What are the risks? How have I been doing on this medication for a period of time? The dashboard's helping me figure that out as well." (P172) *
	**Focus of Consultation**	Structuring visits [Facilitator]	*Q2.9. "It gave us a focus. Sometimes we'll go off on tangents that might not necessarily be productive for my health discussion. The dashboard keeps it on target." (P471) *
**Clinician**	**Supportive Screening Tool **	Consistent monitoring [Facilitator]	*Q3.1. "I get better treatment because she is able to access... the information is right in front of her… I think it's more beneficial for the doctor as far as her being able to recall things, you know, testing and things like that." (P118) *
	**Work Efficiency**	Faster access to patient data [Facilitator]	*Q3.2. "It is a great tool for the doctor, and I get the benefits of the application because the doctor has faster access to the information." (P118)*
**Clinic**	**Quality of Care**	Trust in the division [Facilitator]	*Q4.1. "It showed professionalism and gave me more confidence in the division, you know, that they're capturing data. They're looking at the data in the right way. There's continuity." (P437) *
	**RA Outcome Measures Workflow**	Inconsistent collection of RA outcome measures [Barrier]	*Q4.2. "So, I mean, I would think that if there was a way to consistently do that each time, that would probably be good." (P715) *
		Communication of RA outcome measures by Mas [Barrier]	*Q4.3."There's no discussion in advance with what's in the form. Occasionally, I will also be asked to fill out a form upon arrival at the clinic, and there's usually not much discussion about that either." (P197)*

Regular RA outcome measure assessment and visualization of the dashboard’s graphs showing disease trajectory helped patients understand trends in their DA and PF in relation to medications prescribed. While some patients found this motivating and encouraged adherence to their treatment plan when symptoms improved over time (Table 3.: Q1.4), others found it distressing when target scores were not achieved, or symptoms worsened (Table 3.: Q1.5).

Perceived reliability of RA outcome questionnaires was a third theme generated at the individual level pertaining to patients’ perceived limitations of outcome measures themselves. A few patients considered numeric scales used to measure RA outcomes as inaccurate, given that RA symptoms including arthritis pain fluctuate over time (Table 3.: Q1.6). Others felt the scales were too subjective, particularly when quantifying their symptoms with numeric ratings (Table 3.: Q1.7). They stated that these scales did not objectively capture the nuances of their RA experiences and that the interpretation of symptoms could vary significantly between individuals. One patient reported having difficulty answering questions on the PROMIS-PF questionnaire, citing that the statements were unclear, vague, or irrelevant to their age or medical condition (Table 3.: Q1.8).

#### Interpersonal level

At the interpersonal level, three themes were generated: patient-clinician communication, patient involvement in care, and focus of the clinical visit. In terms of patient-clinician communication, patients felt that using the RA dashboard to discuss outcome measures enhanced their understanding of their clinician’s perception of their disease, especially in cases where there was discordance between patient and clinician RA outcome scores (Table 3., Q:2.1). Further, patients who reported that their clinician collected, reviewed, and provided a thorough explanation of RA outcome scores at every visit were receptive to the dashboard and expressed a desire to review it with their clinician regularly. They considered RA outcome scoring and target measures (specifically CDAI) to be important topics of discussion during clinical visits (Table 3., Q:2.2). Conversely, a few patients reported that their clinician provided less detailed explanations of their RA outcome scores and quickly shared the dashboard with them during visits. They expressed frustration and confusion about the dashboard in general and usability of RA outcomes more specifically (Table 3., Q:2.3). Several patients felt that the use of the RA PRO dashboard helped to initiate new topics of discussion not usually asked about by clinicians that extended beyond traditional healthcare topics, such as factors that contributed to disease flares and their resulting effects (inflammatory diets, personal and professional goals and responsibilities, and sleep routines) (Table 3., Q:2.4). However, one patient, who explained that the clinician does not frequently use the dashboard or discuss RA outcomes during clinical visits, stated that these measures added no new information and provided redundant topics of discussion that might have naturally arisen during the usual clinical encounter (Table 3., Q:2.5).

Regarding involvement in their care, patients explained that collecting RA measures and reviewing the dashboard with their clinician prompted them to recall specific elements that triggered their symptoms, facilitating more detailed discussions with their clinician (Table, Q:2.6). Moreover, some patients reported that filling out RA outcome questionnaires encouraged them to reflect more thoughtfully on their symptoms, especially when responding to the pain question (Table 3., Q:2.7). Additionally, patients discussed that the data presented in the dashboard facilitated goal setting and conversations about goals of care with their clinician. These discussions encompassed various aspects such as treatment initiation, modification, medication side effects, DA targets, maintenance of physical functionality and symptom management, including pain control (Table 3., Q:2.8).

Lastly, one participant expressed that completing RA outcome forms prior to the visit and utilizing the dashboard to discuss scores and changes over time facilitated a more structured approach to clinical visits. This process streamlined communication, guided discussions towards pertinent health topics, and fostered productive health conversations (Table, Q:2.9).

#### Clinician level

The clinician level included two themes: screening tool and work efficiency. The majority of patients described the RA PRO dashboard as a supportive screening tool that aided their clinician in visualizing RA outcome scores during clinical visit. They emphasized that the dashboard offered an overarching view of their condition and assisted clinicians in consistently monitoring their DA, PF, and pain over time, enabling them to monitor patient symptoms effectively. This facilitated shared decision-making and improved treatment outcomes. (Table 3., Q:3.1).

Other patients explained that assessing RA outcome data prior to the visit and sharing the dashboard with patients to discuss updated scores might enhance clinicians’ work efficiency. They felt this approach led to more effective visits by enabling clinicians to prepare for consultations and granting them quicker access to patient data (Table 3., Q:3.2).

#### Clinic level

The clinic level includes two themes: quality of care and RA outcome measures workflow. Participants reported both benefits and limitations regarding the collection of RA outcome measures at the clinic. Specifically, patients highlighted the positive impact of collecting RA outcomes and using the dashboard on their perception of the clinic’s quality of care. They emphasized that it built trust in the department, showcased professionalism, and ensured continuity of care and a commitment to monitoring their disease (Table 3., Q:4.1). Despite their appreciation for collecting RA outcomes, many participants expressed a lack of interest in these measures due to the data collection process employed by the clinic. Some noted that the irregularity in collecting RA outcomes at each visit had a negative impact on their perception of the benefits and utility of these measures by their clinician. As a result, they requested more consistent surveying at each visit to enhance their engagement with the process (Table 3., Q:4.2). Finally, many patients considered completing RA outcome measures as an additional burden, especially when they did not receive explanations from the medical support staff upon receiving the forms. They particularly noted the lack of information regarding the measures being collected, their purpose, use, and benefits, which contributed to their perception of the process as burdensome (Table 3., Q:4.3).

## Discussion

In this study, we used a mixed-methods approach incorporating both surveys and interviews to evaluate patient perceptions of a new health Information Technology (IT) tool that displays RA outcome measures in routine clinical practice. Guided by the EMH as an analysis framework, both the quantitative and qualitative components of the study found that patients endorsed and valued the use of the RA dashboard during clinic visits. In general, they viewed the RA PRO dashboard as a valuable visual tool that enhanced their understanding of their disease and assisted their physicians in gaining a clearer understanding of their priorities The interviews highlighted several key benefits of the dashboard, including its role in enhancing patients’ knowledge, facilitating communication about RA, medications, and other disease-related topics with their clinicians, improving clinical encounters by fostering patient involvement in RA care, and bolstering trust in clinicians and the clinic. Interviews underscored the significance patients attributed to RA outcome measures, emphasizing their view that routine assessment and use of these measures were crucial to RA care, as the questionnaires prompted them to reflect on their health. Although generally positive, both sources of data suggested that some but not all patients believed in the ability of the dashboard to improve inter-personal communication.

Our findings are consistent with the results of other studies assessing the positive impacts of dashboards on patient knowledge, shared decision-making, and patient-clinician communication in the fields of rheumatology, endocrinology, oncology, and psychiatry [17–19, 24, 36]. These patient-facing dashboards have been shown to ()facilitate patient communication with clinicians making it easier to discuss health concerns, treatment options, and outcomes. Our findings expand upon these findings, as patients stated that the RA PRO dashboard is not merely a tool for providing clinicians with easier and faster access to their RA outcomes data but empowers them to analyze and use their own data to guide self-management. Our findings also align with prior qualitative studies, which emphasized that patients value clear communication about the purpose of PROs, their integration in consultations, and guidance on how to complete PRO forms correctly [37].

Despite interest in the dashboard, our study highlights several barriers related to the logistics of collecting RA outcome measures. In line with previous studies, we found that inconsistencies in guidance on administering RA outcome questionnaires [38] and inadequate explanations about the importance of reporting these measures may reduce their perceived usefulness, hinder patient acceptance and engagement and lead to lower PRO completion rates due to respondent burden [39–42]. Addressing this challenge may require a streamlined workflow for the routine collection of RA outcomes, alongside continuous monitoring of adherence to the workflow, including front desk staff, MAs, nurses, and clinicians. Providing comprehensive training for medical support staff can enhance their understanding of the significance of outcome measures in RA care and improve their ability to communicate effectively with patients when administering questionnaires. Additional barriers identified in the study were associated with specific questions included in the RA outcome measure questionnaires, such as perceptions that some of the questions were irrelevant or unclear or lack of specificity in response options tailored to patients' symptoms [37, 43, 44]. These findings indicate that while the dashboard itself is seen as valuable, clinic workflow challenges for collecting the information and the burden of data collection for patients are barriers that require attention [45, 46]. Clear and standardized explanations of the outcome measure forms distributed to patients during check-in for clinical visits, including their purpose and significance in RA care, can also enhance patient understanding and engagement.

We did not find major differences in the quantitative survey results for patients with repeated surveys (and therefore likely more exposures to the RA PRO dashboard). However, in the qualitative interviews, we observed that clinicians’ perceived attitude toward RA outcome measures impacted patients' acceptance and perceived benefits of the dashboard. For instance, patients who received detailed explanations and feedback from their clinicians on their RA outcome scores and those who saw the dashboard at nearly every clinical visit generally showed more interest in seeing the dashboard in future visits. They also expressed higher awareness and knowledge about the importance of collecting and using these measures in RA care. Conversely, patients who reported that their clinicians did not discuss the RA outcome measures or share the dashboard with them expressed more barriers in understanding these measures and limited interest in seeing the dashboard at future visits. It is likely that limited communication with patients about RA outcome scores not only affects their understanding, but also undermines their willingness to engage in the collection of RA outcome measures. Clinician factors related to collection of RA outcomes, including concerns about the potential of the dashboard to lengthen visits, have been explored in our prior work [25]. These findings suggest that integrating the collection and use of RA outcome measures into routine practice, educating clinicians on the value of RA outcome data, and training them on how to interpret and discuss these measures with patients can help normalize their use and emphasize their importance in patient care [47]. Additionally, providing opportunities for patients to ask questions or seek clarification about RA outcome measure forms can further improve their perception of the relevance and utility of these measures in managing RA more effectively.

A key strength of this mixed-methods study was its use of the EHM as a framework, which allowed us to categorize the patients’ perceived benefits and limitations. Despite its strengths, this study is not without limitations. One limitation of this study is the potential for selection bias. The participants who engaged in the study may not be representative of the broader population of RA patients, particularly due to a majority of the interview participants having high levels of education and health literacy. High digital literacy may have biased the study toward positive perspectives of the dashboard. Since all patients included in the study had seen the dashboard at least once during clinical visits, our findings cannot be generalized to those who have never seen the dashboard before. Although we included a range of usability perspectives, the qualitative sample consisted of patients willing to participate in the interview process, which may not have captured the full range of patient views on outcome measures and usability of the RA PRO dashboard in clinical care. Our qualitative sample was small, and may not have captured all possible perspectives. Additionally, since this study was conducted at a single academic medical center, our results may not generalize to other settings.

## Conclusions

In conclusion, this study highlights that most patients found an EHR-integrated RA dashboard that displays their disease outcomes over time useful for improving understanding of their disease, enhancing communication with their clinicians, aiding with shared decision-making, and involving them in care. Future work should test and develop methods to address challenges related to regular collection of data, and to the use and discussion of outcome measures by clinicians.

## Supplementary Information

Supplementary Material 1.

Supplementary Material 2.

Supplementary Material 3.

Supplementary Material 4.

Supplementary Material 5.

Supplementary Material 6.

## Data Availability

De-identified data that support the findings of this study are available on request from the corresponding author. The data are not publicly available due to privacy and ethical restrictions.

## Abbreviations

RA: Rheumatoid Arthritis
DA: Disease Activity
PF: Physical Function
ACR: American College of Rheumatology
PRO: Patient-Reported Outcomes
EMH: Ecological Model of Health
CDAI: Clinical Disease Activity Index
PROMIS-PF: Patient-Reported Outcomes Measurement Information System Physical Function
EHR: Electronic Heath Record
ICD: International Classification of Diseases
MA: Medical Assistant
COREQ: Consolidated Criteria for reporting Qualitative Research
IT: Information Technology
Q: Quote
