# Supplementary material for: Patient perceptions of an electronic-health-record-based rheumatoid arthritis outcomes dashboard: a mixed-methods study
Source: BMC Med Inform Decis Mak. 2024 Oct 12;24:302. doi: 10.1186/s12911-024-02696-9 (PMC11470722; doi:10.1186/s12911-024-02696-9)
Supplement: Supplementary file 2 — Supplementary Material 2. [file 12911_2024_2696_MOESM2_ESM.docx]

**Appendix A: Patient Semi-Structured Interview Guide**

I would like to ask you questions about yourself and your experience with the RA PRO dashboard *[SHARE DASHBOARD SCREENSHOT WITH PATIENT]*

1. When you think about your rheumatoid arthritis, what is the first thing that comes to your mind? Please explain.
   1. What concerns you most about your RA?
2. What type of information do you expect to see or learn about during your appointment/clinic visit with your RA physician? Please explain.
3. Thinking about the RA PRO dashboard that your RA physician used and shared with you during your appointment/clinic visit...
   1. What do you think is the main purpose of this dashboard?
   2. What type of information did your physician discuss when sharing the dashboard with you during your appointment/clinic visit? Please explain.
      1. How did your conversation with your physician change during your appointment/visit before and after viewing or discussing the dashboard? Please explain.
   3. When does your physician usually show/share the dashboard with you? Please explain. *(Beginning, middle, or end of the visit)*
   4. *(For Telehealth visit, ask this additional question)* How does your physician share/discuss the dashboard with you during telehealth visits?
   5. What are the things that you like about the dashboard? *(Design, format, content)*
   6. What are the things that you don’t like or do not find useful about the dashboard? *(Design, format, content)*
   7. What type of changes to you suggest to improve the RA PRO dashboard? Please explain.
4. Is there anything else related to the RA PRO dashboard that you would like to share with me?

Thank you very much for your time and cooperation.
