# Supplementary material for: Patient perceptions of an electronic-health-record-based rheumatoid arthritis outcomes dashboard: a mixed-methods study
Source: BMC Med Inform Decis Mak. 2024 Oct 12;24:302. doi: 10.1186/s12911-024-02696-9 (PMC11470722; doi:10.1186/s12911-024-02696-9)
Supplement: Supplementary file 3 — Supplementary Material 3. [file 12911_2024_2696_MOESM3_ESM.docx]

**Appendix B: Appointment Characteristics of Study Participants**

|  | **Patient Survey Respondents (N=173)** | **Patient Interview Participants (n=29)** |
| --- | --- | --- |
| **Visit Type*, n (%)** |  |  |
| In-person | 154 (89.0) | 26 (89.7) |
| Telehealth | 19 (10.9) | 3 (10.3) |
| **Survey Type**, n (%)** |  |  |
| In-person | 140 (80.9) | 25 (86.2) |
| Phone | 33 (19.1) | 4 (13.8) |
| **Treating Clinician***, n (%)** |  |  |
| Clinician A | 57 (32.9) | 9 (31.0) |
| Clinician B | 31 (17.9) | 4 (13.8) |
| Clinician C | 29 (16.7) | 5 (17.2) |
| Clinician D | 12 (6.9) | 3 (10.3) |
| Clinician E | 11 (6.3) | 3 (10.3) |
| Clinician F | 9 (5.2) | 0 (0) |
| Clinician G | 5 (2.8) | 1 (3.4) |
| Clinician H | 5 (2.8) | 2 (6.9) |
| Clinician I | 3 (1.7) | 1 (3.4) |
| Clinician J | 3 (1.7) | 1 (3.4) |
| Clinician K | 2 (1.1) | 0 (0) |
| Clinician L | 2 (1.1) | 0 (0) |
| Clinician M | 2 (1.1) | 0 (0) |
| Clinician N | 1 (0.5) | 0 (0) |
| Clinician O | 1 (0.5) | 0 (0) |

*Visit Type corresponds with their initial survey date.
**Survey Type corresponds with their initial survey date.
***Treating Clinician corresponds with their initial survey date.
