# Supplementary material for: Patient perceptions of an electronic-health-record-based rheumatoid arthritis outcomes dashboard: a mixed-methods study
Source: BMC Med Inform Decis Mak. 2024 Oct 12;24:302. doi: 10.1186/s12911-024-02696-9 (PMC11470722; doi:10.1186/s12911-024-02696-9)
Supplement: Supplementary file 4 — Supplementary Material 4. [file 12911_2024_2696_MOESM4_ESM.docx]

**Supplementary Material: Summary of Initial and Most Recent Survey Responses among Participants with >1 Survey Response, categorized according to the Ecological Model of Health (n=57)**

|  | **First Survey** | | | | **Last Survey** | | | |
| --- | --- | --- | --- | --- | --- | --- | --- | --- |
| **INDIVIDUAL, n (%)** | **Yes** | **Some- what** | **No** | **Missing/Unsure** | **Yes** | **Some- what** | **No** | **Missing/Unsure** |
| Would you like to see the dashboard again at your next visit? | 52 (91.2) | - | 3 (5.3) | 2 (3.5) | 47 (82.5) | - | 4 (7.0) | 6 (10.5) |
| Did the dashboard help you understand more about your RA? | 48 (84.2) | 9 (15.8) | 0 (0) | 0 (0) | 45 (79.0) | 9 (15.8) | 3 (5.3) | 0 (0) |
| Did the dashboard help you understand more about why you take certain medicines? | 37 (64.9) | 6 (10.5) | 10 (17.5) | 4 (7.0) | 34 (59.6) | 13 (22.8) | 8 (14.0) | 2 (3.5) |
| Did the dashboard help you share information about your RA with other people (such as family members, friends, or other healthcare providers)? | 26 (45.6) | 9 (15.8) | 12 (21.1) | 10 (17.5) | 26 (45.6) | 6 (10.5) | 13 (22.8) | 12 (21.1) |
| **INTERPERSONAL, n (%)** | **Yes** | **Some-** **what** | **No** | **Missing/Unsure** | **Yes** | **Some-** **what** | **No** | **Missing/Unsure** |
| Did the dashboard help you talk to your doctor about your RA or your symptoms? | 45 (78.9) | 6 (10.5) | 5 (8.8) | 1 (1.8) | 44 (77.2) | 9 (15.8) | 3 (5.3) | 1 (1.8) |
| Did the dashboard help you talk to your doctor about your medicines? | 44 (77.2) | 7 (12.3) | 3 (5.3) | 3 (5.3) | 39 (68.4) | 10 (17.5) | 7 (12.3) | 1 (1.8) |
| Did the dashboard help you make better decisions about your RA care? | 38 (66.7) | 10 (17.5) | 5 (8.8) | 4 (7.0) | 42 (73.7) | 8 (14.0) | 4 (7.0) | 3 (5.3) |
| Did the dashboard help you talk about things that are important to managing your disease, other than your medicines? | 38 (66.7) | 5 (8.8) | 10 (17.5) | 4 (7.0) | 38 (66.7) | 8 (14.0) | 11 (19.3) | 0 (0) |
| Do you think using the dashboard helped your communication with your doctor? | 36 (63.2) | 12 (21.1) | 6 (10.5) | 3 (5.3) | 34 (59.6) | 14 (24.6) | 6 (10.5) | 3 (5.3) |
| Do you think using the dashboard changed the focus of your visit? | 20 (5.1) | 12 (21.1) | 19 (33.3) | 6 (10.5) | 13 (22.8) | 12 (21.1) | 28 (49.1) | 4 (7.0) |
| **CLINICIAN, n (%)** | **Yes** | **Some-** **what** | **No** | **Missing/Unsure** | **Yes** | **Some-** **what** | **No** | **Missing/Unsure** |
| Do you think using the dashboard helped your doctor to better understand what's most important to you? | 25 (43.9) | 10 (17.5) | 9 (15.8) | 12 (21.1) | 28 (49.1) | 13 (22.8) | 10 (17.5) | 6 (10.5) |
| Do you think using the dashboard gave your doctor information about you that s/he may not have gotten without the dashboard? | 23 (40.4) | 11 (19.3) | 11 (19.3) | 12 (21.1) | 23 (40.4) | 13 (22.8) | 13 (22.8) | 8 (14.0) |
