# Supplementary material for: Patient perceptions of an electronic-health-record-based rheumatoid arthritis outcomes dashboard: a mixed-methods study
Source: BMC Med Inform Decis Mak. 2024 Oct 12;24:302. doi: 10.1186/s12911-024-02696-9 (PMC11470722; doi:10.1186/s12911-024-02696-9)
Supplement: Supplementary file 5 — Supplementary Material 5. [file 12911_2024_2696_MOESM5_ESM.docx]

**Supplementary Material: Socio-demographic Characteristics of Patients that were Invited to Participate in a Patient Interview (n=141)**

|  | **Patients Invited to Participate**  **in Patient Interviews (n=141)** |
| --- | --- |
| **Age, mean (SD)** | 59.6 (14.5) |
| <30 | 1 (0.7) |
| 30-44 | 28 (19.9) |
| 45-64 | 46 (32.6) |
| 65-74 | 46 (32.6) |
| ≥75 | 20 (14.2) |
| **Gender, n (%)** |  |
| Female | 123 (87.2) |
| Male | 18 (12.8) |
| **Race and Ethnicity,** **n (%)** |  |
| African American or Black | 14 (9.9) |
| Asian | 26 (18.4) |
| Hispanic | 19 (13.5) |
| Mixed/Other | 6 (4.3) |
| White | 73 (51.8) |
| Unknown | 3 (2.1) |
| **Insurance,** **n (%)** |  |
| Medicaid | 17 (12.1) |
| Medicare | 73 (51.8) |
| Private/Commercial | 51 (36.2) |
| **Baseline Comorbidities*, n (%)** |  |
| Anxiety | 20 (14.2) |
| Depression | 17 (12.1) |
| Fibromyalgia | 8 (5.7) |
| **Baseline RA Outcomes**** |  |
| CDAI, n | 133 |
| median (IQR) | 8.5 (4.7 - 16.3) |
| PROMIS, n | 124 |
| median (IQR) | 42.6 (36.0 - 49.1) |
| Pain, n | 118 |
| median (IQR) | 32 (13.0 - 59.0) |

*Baseline Comorbidities defined as 1 ICD code within 12 months prior to their clinic visit date.
**Baseline RA Outcomes were the most recent RA outcome within 18 months to their clinic visit date.
