# Supplementary material for: Patient perceptions of an electronic-health-record-based rheumatoid arthritis outcomes dashboard: a mixed-methods study
Source: BMC Med Inform Decis Mak. 2024 Oct 12;24:302. doi: 10.1186/s12911-024-02696-9 (PMC11470722; doi:10.1186/s12911-024-02696-9)
Supplement: Supplementary file 6 — Supplementary Material 6. [file 12911_2024_2696_MOESM6_ESM.docx]

**Supplementary Material: Summary of Initial Survey Responses among Qualitative Interview Participants, categorized according to the Ecological Model of Health (n=29)**

| **INDIVIDUAL, n (%)** | **Yes** | **Somewhat** | **No** | **Missing/Unsure** |
| --- | --- | --- | --- | --- |
| Would you like to see the dashboard again at your next visit? | 25 (86.2) | - | 1 (3.4) | 3 (10.3) |
| Did the dashboard help you understand more about your RA? | 19 (65.5) | 8 (27.6) | 1 (3.4) | 1 (3.4) |
| Did the dashboard help you understand more about why you take certain medicines? | 14 (48.3) | 4 (13.8) | 10 (34.5) | 1 (3.4) |
| Did the dashboard help you share information about your RA with other people (such as family members, friends, or other healthcare providers)? | 12 (41.4) | 0 (0) | 10 (34.5) | 7 (24.1) |
| **INTERPERSONAL, n (%)** | **Yes** | **Somewhat** | **No** | **Missing/Unsure** |
| Did the dashboard help you talk to your doctor about your RA or your symptoms? | 21 (72.4) | 6 (20.7) | 1 (3.4) | 1 (3.4) |
| Did the dashboard help you talk to your doctor about your medicines? | 19 (65.5) | 5 (17.2) | 3 (10.3) | 2 (6.9) |
| Did the dashboard help you make better decisions about your RA care? | 19 (65.5) | 5 (17.2) | 4 (13.8) | 1 (3.4) |
| Did the dashboard help you talk about things that are important to managing your disease, other than your medicines? | 18 (62.1) | 3 (10.3) | 8 (27.6) | 0 (0) |
| Do you think using the dashboard helped your communication with your doctor? | 16 (55.2) | 7 (24.1) | 4 (13.8) | 2 (6.9) |
| Do you think using the dashboard changed the focus of your visit? | 5 (17.2) | 8 (27.6) | 14 (48.3) | 2 (6.9) |
| **CLINICIAN, n (%)** | **Yes** | **Somewhat** | **No** | **Missing/Unsure** |
| Do you think using the dashboard helped your doctor to better understand what's most important to you? | 9 (31.0) | 7 (24.1) | 7 (24.1) | 6 (20.7) |
| Do you think using the dashboard gave your doctor information about you that s/he may not have gotten without the dashboard? | 8 (27.6) | 6 (20.7) | 7 (24.1) | 8 (27.6) |
